# Aggressive refractory pemphigus vulgaris that responded to plasmapheresis: a case report

**DOI:** 10.1186/s13256-020-02421-w

**Published:** 2020-07-19

**Authors:** Hiba Hasan Khaddour, Diana Zaher, Triak Kassem, Ahmad Hasan

**Affiliations:** Department of Dermatology, Tishreen Hospital, Damascus, Syria

**Keywords:** Refractory pemphigus vulgaris, Therapeutic plasma exchange, Plasmapheresis, Case report

## Abstract

**Background:**

Pemphigus vulgaris is an autoimmune disorder that involves intraepithelial blistering and sores of the skin and mucous membranes. The average age of onset is between 50 and 70 years. Pemphigus rarely occurs in children. It correlates with the level of circulating autoantibodies; therapeutic plasma exchange is hypothesized to remove pathogenic autoantibodies, and this is necessary in refractory severe cases.

**Case presentation:**

A 14-year-old Asian girl came to our hospital with blisters and erosions all over her body and in the oral mucosa. She was diagnosed with pemphigus vulgaris by skin biopsy about 3 months before hospitalization. She was admitted to the intensive care unit due to aggressively worsening symptoms, extensive lesions, dehydration, and electrolyte imbalance secondary to excess fluid loss from the skin wounds and sepsis secondary to infection of the exposed wounds. She did not respond to prednisone and azathioprine therapy but was successfully treated with plasmapheresis.

**Conclusion:**

The purpose of this case report is to describe an aggressive presentation of pemphigus vulgaris, especially because the onset of the disease in our patient was at an early age. The disease rarely begins in childhood, and this case report highlights the importance of plasmapheresis as a useful intervention in patients with pemphigus vulgaris who are not responding to conventional therapy, taking into account that there is a paucity of studies showing the effectiveness of plasmapheresis in inducing partial or complete remission in young patients.

## Background

Pemphigus vulgaris (PV) encompasses a group of life-threatening autoimmune bullous diseases characterized by flaccid blisters and erosions of the mucous membrane and skin. The average age of onset is between 50 and 70 years. PV rarely occurs in children [1]. The severity of the disease is based on its progressive course, which is accompanied by increased body catabolism with loss of body fluids and proteins and secondary bacterial infections that may lead to sepsis and cardiac failure [2]. The mainstay of treatment remains corticosteroids with or without adjuvant therapy. Adjuvant therapy includes steroid-sparing agents and immunotherapy procedures. Due to the variation in severity, varied response to the conventional treatment protocols, and severe side effects, dermatologists all over the world have ventured into using new modalities of adjuvant therapies, such as intravenous immunoglobulins and therapeutic plasma exchange, to grapple the increasing number of patients with severe PV who show little or no response to conventional corticosteroid treatment [3]. We report a case of severe PV in a patient who did not respond to prednisone and azathioprine therapy but was successfully treated with plasmapheresis.

## Case presentation

### Patient information

A 14-year-old Asian girl came to our hospital with blisters and erosions all over her body and in the oral mucosa. She had been diagnosed with PV by a skin biopsy about 3 months before hospitalization and had been treated with 1 mg/kg/day of oral prednisone. She had no history of diabetes mellitus, cardiovascular diseases, or psoriasis; no psychosocial history; and no familial history of pemphigus diseases. Her parents are not biologically related. The severity of the disease led to the following life-threatening issues: dehydration and electrolyte imbalance secondary to excess fluid loss from the skin wounds, as well as sepsis secondary to infection of the exposed wounds. Due to progressively worsening symptoms, extensive lesions, and high susceptibility to further infections, the patient was admitted to the intensive care unit. The following immediate actions were taken: urinary catheterization and resuscitation with intravenous fluids to correct the circulatory and electrolyte imbalance, early management of sepsis with intravenous antibiotics, and proper pain relief with opioids. The differential diagnosis included allergy eruption [4], staphylococcal scalded skin syndrome, and Stevens-Johnson syndrome.

### General physical examination on admission

The patient was depressed and slightly dull. Her vital signs were as follows; temperature 38.5 °C, blood pressure 90/60 mmHg, heart rate 115 beats/minute, and respiratory rate 24 breaths/minute. Her mucosae were pale. Her capillary refill time was < 2 seconds. Her musculoskeletal system was normal. Her lymph node examination revealed no findings. Her cardiac examination revealed tachycardia with no murmur. Her lung examination revealed no rales or wheeze. Her mental status (consciousness) was slightly obtunded. The result of her cranial nerve examination was normal. Her pupils were equal with normal direct and indirect pupillary light reflexes. Her motor examination revealed muscle bulk and tone were normal. Her strength was full bilaterally. Her reflexes were normal and symmetric at the biceps, triceps, knees, and ankles. Her plantar responses were flexor. Her sensory examination revealed that her light touch, pinprick, position sense, and vibration sense were normal in her fingers and toes.

### Clinical cutaneous findings

**Fig. 1:**
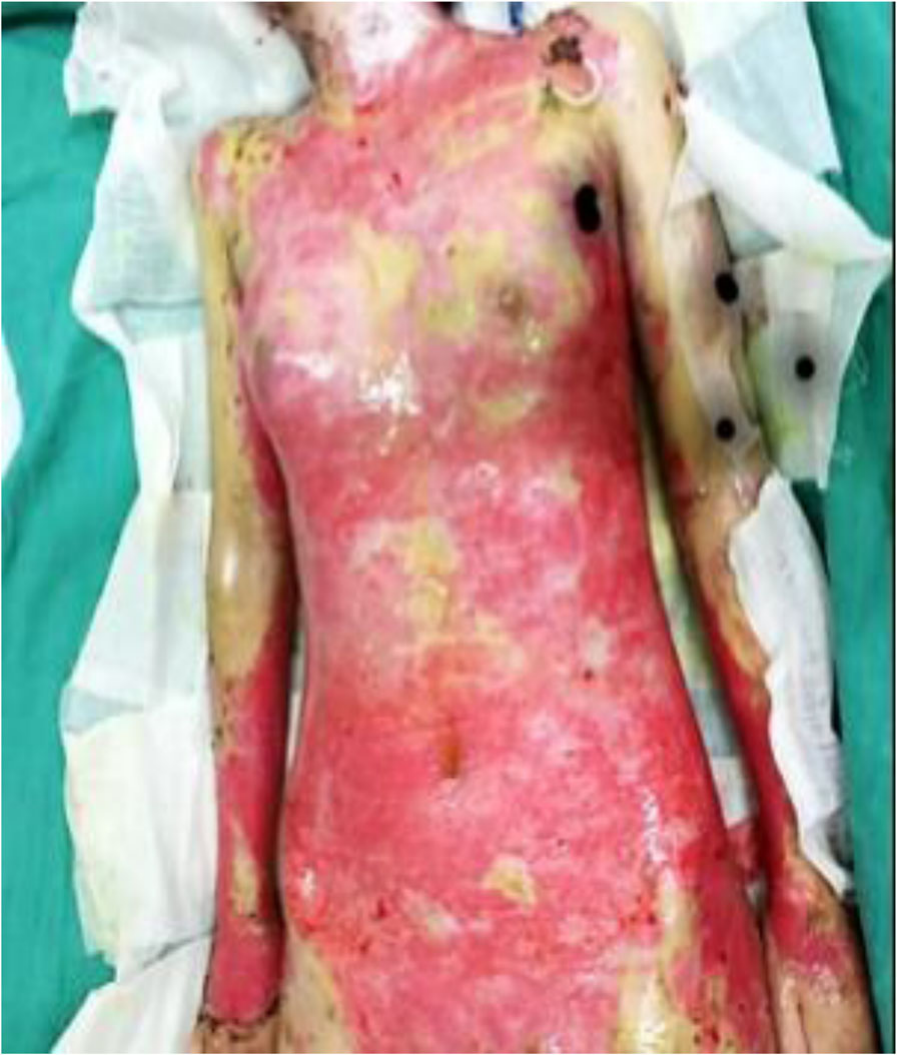
Flaccid blisters and erosions involving more than 75% of the body surface area (face, trunk, and limbs)

**Fig. 2:**
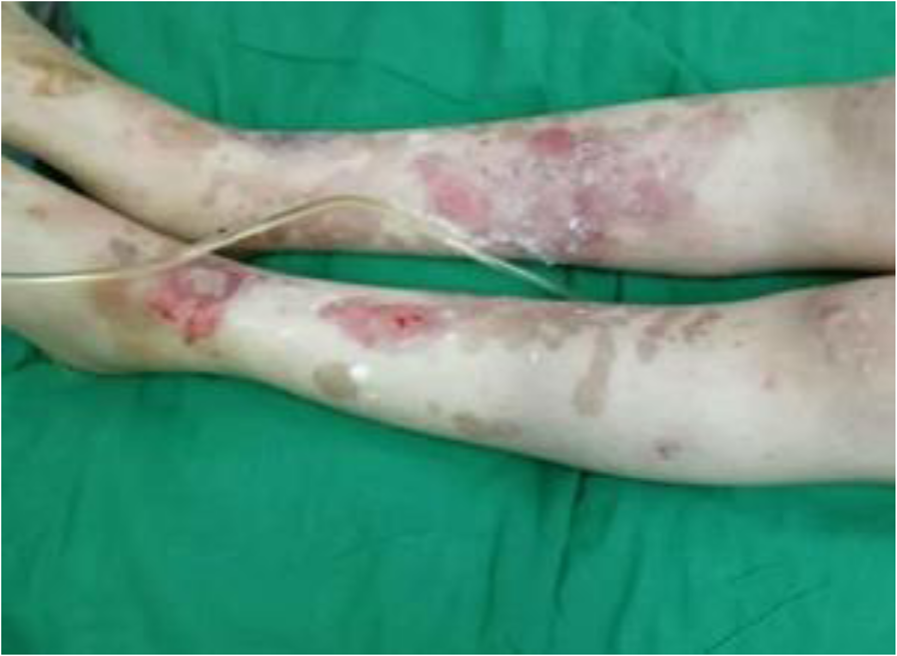
Flaccid blisters on the legs and oozing from skin ulcerations

The patient was febrile with numerous flaccid blisters and erosions involving the oral mucosa and more than 75% of the body surface area (face, trunk, and limbs), some covered with slough (Figs. 1 and 2). She had oozing from ulceration and hemorrhagic excoriation with the peeling of the skin. Her perilesional Nikolsky sign was positive. The rest of her physical examination was normal.

### Diagnostic testing

**Fig. 3:**
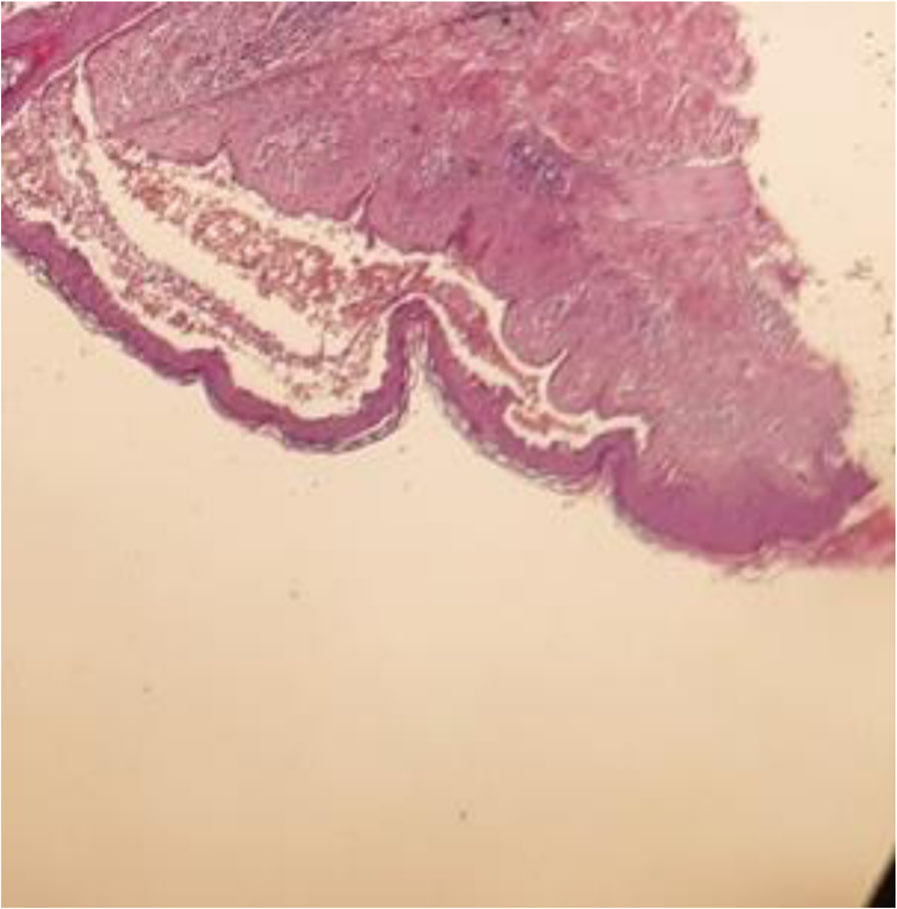
Histopathology confirmed acantholysis in the epidermis with intraepithelial blistering

Skin biopsy confirmed the diagnosis of PV. Histology showed separated keratinocytes (acantholytic cells) just above the basal layer of the epidermis (Fig. 3).

### Blood analysis

The patient’s complete blood count revealed the following values: white blood cells, 18,000 C/UL 18 x 10^9^/L; neutrophils, 86%; lymphocyte count, 10%; hemoglobin, 10 mg/dl 100g/L; hematocrit, 30.3% 0.303 proportion of 1; mean corpuscular volume, 79.5 fl; mean corpuscular hemoglobin, 22.8 pg; platelets, 150 C/UL 150 x 10^9^/L; alanine aminotransferase, 16.3 U/L; aspartate aminotransferase, 53.9 U/L; total bilirubin, 4.3 μmol/L; lactate dehydrogenase, 408 U/L; erythrocyte sedimentation rate, 27 mm/hour; C-reactive protein, 48 mg/L; blood urea nitrogen, 2.8 mmol/L; creatinine, 50.8 μmol/L; potassium, 3.5 mmol/L; and sodium, 136.4 mmol/L. Urinalysis showed no infection. Serum antibodies could not be measured, because the patient could not afford the test.

### Microbiology

At the time of admission, two blood cultures and many swabs from the erosions on the patient’s trunk and limbs were collected for culture. The cultures were processed in the laboratory as per standard microbiological techniques and were cultured for aerobic and anaerobic bacteria. The organism was an encapsulated, gram-negative, rod-shaped bacterium. The isolates were identified as *Pseudomonas aeruginosa*. Blood culture and the pus culture from the erosions revealed the presence of *P. aeruginosa*. The organism was found to be sensitive to polymyxin B or colistin (colistimethate sodium).

### Therapeutic intervention

The patient was treated with oral prednisolone at a dose of 1 mg/kg/day with oral azathioprine at a dose of 150 mg/day (3 mg/kg/day) on admission for 7 days and intravenous ceftazidime 1 g three times per day + oral levofloxacin 500 mg once per day. Treatment with these antibiotics continued until the results of blood and pus cultures were obtained. The bullous skin lesions had breakdown, leaving painful, eroded areas of skin with a similar appearance to the type of wounds found on burn victims. Early assessment by the dermatologists and plastic surgery team resulted in the placement of appropriate dressings to protect the skin wounds and relieve the patient’s distress. One week after the treatment, she did not show any improvement, and she developed fever spikes every day. Blood culture and pus culture from the erosions revealed the presence of *P. aeruginosa*. On the basis of sensitivity reports, she was started on intravenous colistin (colistimethate sodium) 9 mIU/day in three divided doses for 10 days. Because the disease remained active, the prednisolone dose was increased to 1.5 mg/kg/day 3 days after the start of antibiotics. Ten days after that, the erosions persisted but showed slight evidence of re-epithelialization. The patient’s Nikolsky sign was still positive, and she continued to develop a few new blisters. She also developed edema of the genitalia. Because the disease remained active and she was in sepsis with persistent fever, we could not consider giving her intravenous corticosteroids. Considering all the above factors, she was given four sessions of plasmapheresis over 10 days with a gap of 2 days between the sessions. Two liters of plasma were removed using a plasma filter attached to the dialysis machine and replaced with 1 L of normal saline and 1 L of fresh frozen plasma. The procedure was done by a nephrologist in the nephrology department of Tishreen Hospital. After the fourth session of plasmapheresis, the patient’s Nikolsky sign became negative, and no new blisters appeared. The erosions showed re-epithelialization. Serum antibody levels before and after plasmapheresis sessions could not be measured, because the patient could not afford the test.

### Follow-up

**Fig. 4:**
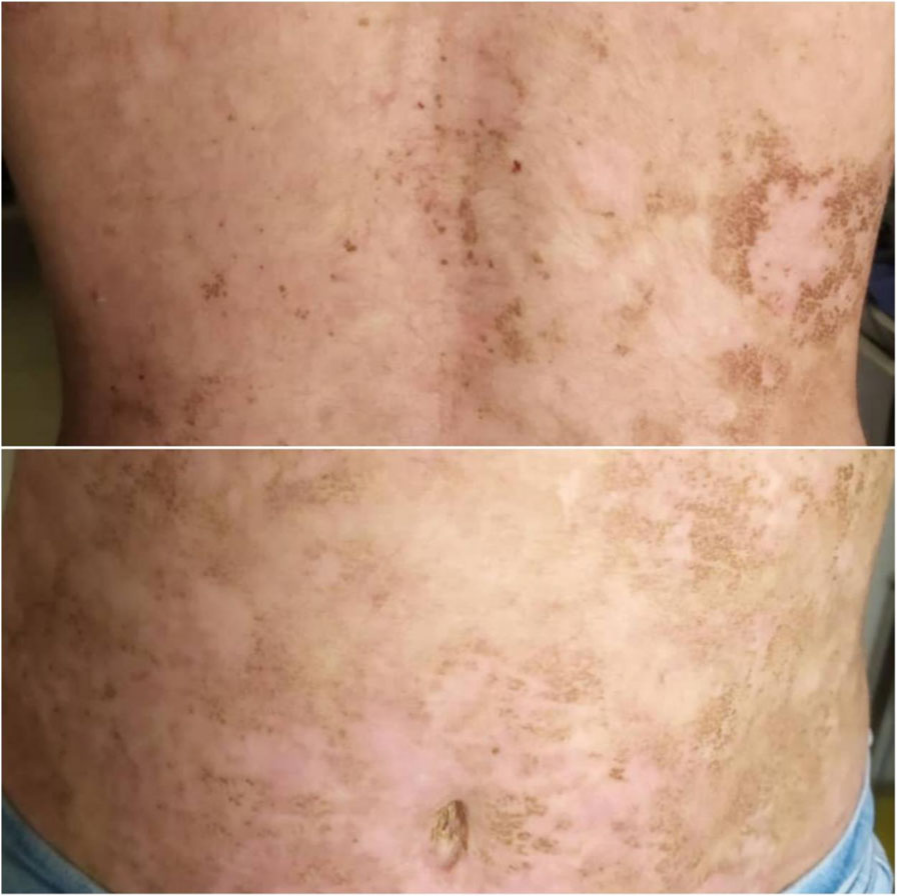
Follow-up: around 90% of erosions healed after 1 month of plasmapheresis

**Fig. 5:**
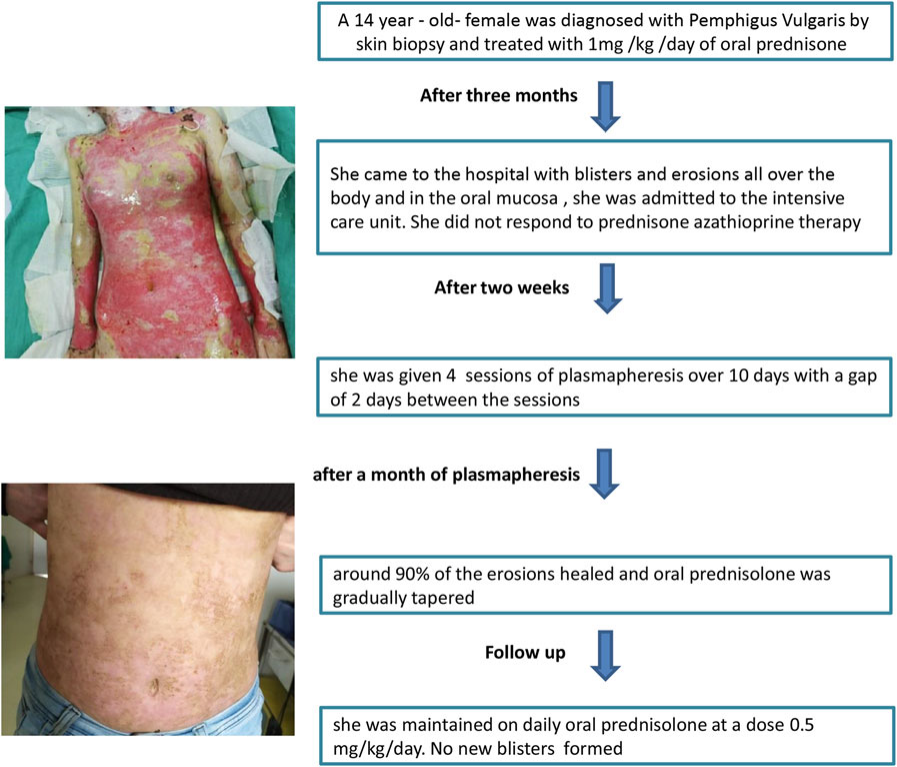
Timeline

Around 90% of the erosions healed after 1 month of plasmapheresis (Fig. 4), and oral prednisolone was gradually tapered over 3 months. The patient was maintained on daily oral prednisolone at a dose 0.5 mg/kg/day, and no new blisters formed (Fig. 5).

## Discussion and conclusion

### Summary

A 14-year-old- girl came to our hospital with blisters and erosions all over her body and in the oral mucosa. She was diagnosed with PV by skin biopsy about 3 months before hospitalization. She was admitted to the intensive care unit due to aggressively worsening symptoms, extensive lesions, dehydration, and electrolyte imbalance secondary to excess fluid loss from the skin wounds and sepsis secondary to infection of the exposed wounds. She did not respond to prednisone and azathioprine therapy but was successfully treated with plasmapheresis.

The importance of this case comes from the fact that the average age of onset of PV is between 50 and 70 years, and PV rarely occurs in children. Most studies published in the medical literature have reported patients with PV in the age range of 40–80 years. The unusual occurrence of PV in a girl can be explained by the psychological severity of the dire conditions that the patient has experienced in the area where she lives.

PV is a serious and potentially life-threatening disease. It can be fatal because there is a loss of the epidermal barrier and a loss of body fluids, and there are chances of a secondary infection. Early and accurate diagnosis is a prerequisite for immediate therapy to prevent fatal outcomes. The clinical diagnosis must be confirmed by histopathological evaluation [5].

The use of systemic corticosteroids with or without immunosuppression is the mainstay of treatment of PV. However, there are still some patients who undergo unacceptably high doses of corticosteroids. We cannot taper their high dosages of medications or treat those who with extreme degree of disease [6].

The role of topical corticosteroids is controversial in PV [7]. The presence of circulating antibodies has an essential role in the pathogenesis of PV. Plasmapheresis is a treatment for reducing the titers of circulating autoantibodies, which has correlated with clinical improvement [8]. Combination therapy of plasmapheresis and immunosuppressants appears to lead to complete remission and brings the disease to a level that can be controlled by corticosteroids. In our patient’s case, clinical evaluation was used to monitor the efficacy of treatment, and no adverse effects of plasma exchange have occurred.

Most studies published in the medical literature involving patients with PV and pemphigoid pemphigus have ages between 40 and 80 years. These studies have reported the effectiveness of plasmapheresis by inducing clinical improvement and the possibility of reducing the dose of corticosteroids by a large percentage and showed that the side effects of plasmapheresis are few [9–12].

The purpose of this case report is to describe an aggressive presentation of PV, especially since the onset of the disease was at an early age and the disease rarely begins in childhood. This case highlights the importance of plasmapheresis as a useful intervention in patients with PV who are not responding to conventional therapy, taking into account that there is a paucity of studies that demonstrated the effectiveness of plasmapheresis in inducing partial or complete remission in young patients.

### Patient perspective

The patient is grateful because she is still alive. She hopes that the dose of corticosteroids will be reduced to the lowest possible dose and that the treatment will not be lifelong. She hopes that the disease will not affect her life course, especially because she is still a child, and that doctors will discover a radical treatment for her illness.

## Data Availability

All relevant data and materials are included in this publication.

## Abbreviation

PV: Pemphigus vulgaris
